# Taxogenomic and Comparative Genomic Analysis of the Genus *Saccharomonospora* Focused on the Identification of Biosynthetic Clusters PKS and NRPS

**DOI:** 10.3389/fmicb.2021.603791

**Published:** 2021-03-11

**Authors:** Ninfa Ramírez-Durán, Rafael R. de la Haba, Blanca Vera-Gargallo, Cristina Sánchez-Porro, Scarlett Alonso-Carmona, Horacio Sandoval-Trujillo, Antonio Ventosa

**Affiliations:** ^1^Faculty of Medicine, Autonomous University of the State of Mexico, Toluca, Mexico; ^2^Department of Microbiology and Parasitology, Faculty of Pharmacy, University of Sevilla, Seville, Spain; ^3^Department of Biological Systems, Metropolitan Autonomous University-Xochimilco, Mexico City, Mexico

**Keywords:** actinobacteria, *Saccharomonospora*, biosynthetic gene clusters, secondary metabolites, polyketide synthase, non-ribosomal peptide synthetase, comparative genomic analysis, taxophylogenomic analysis

## Abstract

Actinobacteria are prokaryotes with a large biotechnological interest due to their ability to produce secondary metabolites, produced by two main biosynthetic gene clusters (BGCs): polyketide synthase (PKS) and non-ribosomal peptide synthetase (NRPS). Most studies on bioactive products have been carried out on actinobacteria isolated from soil, freshwater or marine habitats, while very few have been focused on halophilic actinobacteria isolated from extreme environments. In this study we have carried out a comparative genomic analysis of the actinobacterial genus *Saccharomonospora*, which includes species isolated from soils, lake sediments, marine or hypersaline habitats. A total of 19 genome sequences of members of *Saccharomonospora* were retrieved and analyzed. We compared the 16S rRNA gene-based phylogeny of this genus with evolutionary relationships inferred using a phylogenomic approach obtaining almost identical topologies between both strategies. This method allowed us to unequivocally assign strains into species and to identify some taxonomic relationships that need to be revised. Our study supports a recent speciation event occurring between *Saccharomonospora halophila* and *Saccharomonospora iraqiensis*. Concerning the identification of BGCs, a total of 18 different types of BGCs were detected in the analyzed genomes of *Saccharomonospora*, including PKS, NRPS and hybrid clusters which might be able to synthetize 40 different putative products. In comparison to other genera of the *Actinobacteria*, members of the genus *Saccharomonospora* showed a high degree of novelty and diversity of BGCs.

## Introduction

*Actinobacteria* are a diverse group of clinical, industrial and ecologically important bacteria known for their capacity to cause diseases as well as their potential to produce secondary metabolites widely used in a variety of fields, such as medicine, pharmacy, industrial microbiology and biotechnology, among others (Bérdy, 2012; Doroghazi and Metcalf, 2013). Synthesis of bacterial secondary metabolites might have been originated due to necessary adaptations to diverse environments with the objective to help the producer microorganism to compete for survival resources (Doroghazi and Metcalf, 2013).

Genes involved in secondary metabolite production are commonly grouped within the genome and are usually known as biosynthetic gene clusters (BGCs) (Osbourn, 2010). *Actinobacteria* represent one of the most important sources for discovering new biologically active metabolites (Kamjam et al., 2017; Matsumoto and Takahashi, 2017) due to the presence of two main biosynthetic clusters, polyketide synthase (PKS) and non-ribosomal peptide synthetase (NRPS), involved in the synthesis of bioactive molecules through multifunctional pathways (Fischbach and Walsh, 2006). Biosynthetic gene clusters for secondary metabolites have been studied in actinobacteria isolated from different environments, such as soil (Sharma et al., 2016; Wang et al., 2018), caves (Gosse et al., 2019), freshwater (Zothanpuia et al., 2018), marine (Zhang et al., 2019; Ishaque et al., 2020) and as endophytes from plants (Passari et al., 2016, 2017). Halophilic actinobacteria from extreme ecosystems have also been reported, but only biosynthetic gene clusters from *Actinopolyspora erythraea* YIM 90600^T^ have been located and identified so far (Chen et al., 2014; Corral et al., 2020).

Although a large number of secondary metabolites has been discovered, new bioactive products are still required to cover the current needs of clinical, veterinary and agricultural areas, given the frequent development of microbial resistance of pathogens (Rangseekaew and Pathom-aree, 2019), the appearance of novel diseases, the existence of naturally resistant bacteria and the toxicity of several compounds used in agriculture and cattle industry (Fischbach and Walsh, 2009). The rapid advance of complete genome sequencing and comparative genomic techniques have made it possible to apply bioinformatic tools, such as genomic mining, to carry out a comparative analysis of genomes available in public databases (Challis, 2008). This approach allows one, on the one hand, to comprehend the evolutionary relationships among microbial species and, on the other hand, to identify clusters of genes involved in biosynthetic pathways of relevant biomolecules with potential use for disease treatments.

The actinobacterial genus *Saccharomonospora*, belonging to the family *Pseudonocardiaceae*, was proposed in 1971 (Nonomura and Ohara, 1971) and currently comprises 14 validly published species names (Nonomura and Ohara, 1971; Challis, 2008; Syed et al., 2008; Fischbach and Walsh, 2009; Veyisoglu et al., 2013; Chen et al., 2014; Passari et al., 2016, 2017; Tseng et al., 2018; Zothanpuia et al., 2018; Rangseekaew and Pathom-aree, 2019; Zhang et al., 2019; Corral et al., 2020; Ishaque et al., 2020). The species name “*Saccharomonospora internatus*” has been effectively but not validly published (Greiner-Mai et al., 1987) and has been recently reclassified as an additional strain of the species *Saccharomonospora viridis* (Nouioui et al., 2018). Finally, the species initially described as *Saccharomonospora paurometabolica* (Li et al., 2003) was proved to be a subspecies of *Saccharomonospora iraqiensis* (Nouioui et al., 2018). Members of this genus have been isolated from diverse environments, such as soil, lake sediments, marsh soil, peat, manure, compost, and overheated fodder, with some of them being classified as halophilic microorganisms (Kim, 2015).

In this study we have carried out phylogenomic and comparative genomic analyses of the genus *Saccharomonospora* aimed at reviewing the current taxonomy of this genus and locating and identifying PKS and NRPS biosynthetic clusters present in the genomes with potential for future development of novel active biomolecules.

## Materials and Methods

### Genome Sequence Retrieval

All genome sequences affiliated to members of the genus *Saccharomonospora* and publicly available at the National Center for Biotechnology Information (NCBI) and at the Joint Genome Institute (JGI) databases as of May 31st, 2019 were retrieved. A total of 19 complete or draft genomes were recovered, including type and other reference strains (Table 1). Genome sequences for the type strains of the species *S. colocasiae*, *S. oceani*, and *S. xiaoerkulensis* were not included in the analyses due to the absence of data in public databases. Prediction of Open Reading Frames and genome annotation, including protein-coding genes as well as other functional genome units such as structural RNAs, tRNAs, was performed following the NCBI Prokaryotic Genome Annotation Pipeline (PGAP) (Haft et al., 2018).

**TABLE 1:** Main features of genome sequences of strains of the genus *Saccharomonospora* used in this study.

**Strain**	**Accession No.**	**Assembly**	**Level**	**Size (Mb)**	**GC%**	**Scaffolds**	**Contigs**	**CDS**	**N50**	**L50**	**Group**
*Saccharomonospora amisosensis* DSM 45685^T^	PRJNA546941 ^1^	GCF_011761185.1	Scaffold	5.53	68.6	3	3	5,395	4,804,314	1	Marine/Lake
*Saccharomonospora azurea* NA-128^T^	CM001466.1	GCF_000231055.3	Chromosome	4.76	70.0	1	96	4,406	4,763,852	1	Terrestrial
*Saccharomonospora azurea* SZMC 14600	AHBX00000000.1	GCF_000236985.1	Contig	4.97	70.3	216	218	4,624	55,033	29	Terrestrial
*Saccharomonospora cyanea* NA-134^T^	CM001440.1	GCF_000244975.1	Chromosome	5.41	69.7	1	5	5,086	5,408,301	1	Terrestrial
*Saccharomonospora glauca* K62^T^	CM001484.1 (chromosome), CM001485.1 (plasmid)	GCF_000243395.3	Chromosome	4.56	69.1	2	11	4,269	4,538,746	1	Terrestrial
*Saccharomonospora halophila* 8^T^	AICX00000000.1	GCF_000383775.1	Scaffold	3.69	70.4	116	644	3,656	69,647	16	Moderately halophilic terrestrial
*Saccharomonospora iraqiensis* subsp. *iraqiensis* IQ-H1^T^	AICW00000000.1	GCF_000430445.1	Scaffold	3.90	71.0	139	732	4,014	87,719	16	Moderately halophilic terrestrial
*Saccharomonospora iraqiensis* subsp. *paurometabolica* YIM 90007^T^	AGIT00000000.2	GCF_000231035.3	Scaffold	4.67	71.3	82	1,031	4,537	171,692	9	Moderately halophilic terrestrial
*Saccharomonospora marina* XMU15^T^	CM001439.1	GCF_000244955.1	Chromosome	5.97	68.9	1	8	5,664	5,965,593	1	Marine/Lake
*Saccharomonospora piscinae* KCTC 19743^T^	VCEK00000000.1	GCF_005862235.1	Scaffold	4.90	71.0	11	21	4,508	1,086,926	3	Marine/Lake
*Saccharomonospora saliphila* YIM 90502^T^	AICY00000000.1	GCF_000383795.1	Scaffold	4.03	70.6	145	679	3,916	96,274	11	Moderately halophilic terrestrial
*Saccharomonospora viridis* DSM 43017^T^	CP001683.1	GCF_000023865.1	Complete	4.31	67.3	1	1	3,870	4,308,349	1	Clinical
*Saccharomonospora viridis* JCM 3315	JRZE00000000.1	GCF_000787535.1	Contig	4.30	67.4	13	13	3,876	1,283,650	2	Clinical
*Saccharomonospora viridis* ATCC 33517	FOWS00000000.1	GCF_900115515.1	Scaffold	4.31	67.4	12	17	3,879	1,283,650	2	Clinical
*Saccharomonospora xinjiangensis* XJ-54^T^	AICV00000000.1	GCF_000258175.1	Scaffold	4.78	68.9	2	16	4,366	4,773,543	1	Terrestrial
*Saccharomonospora* sp. 31sw	CP038101.1	GCF_004519465.1	Complete	4.71	69.1	1	1	4,350	4,713,652	1	Terrestrial
*Saccharomonospora* sp. LRS4.154	MWIH00000000.1	GCF_002077655.1	Scaffold	4.86	71.0	13	17	4,568	731,563	3	Marine/Lake
*Saccharomonospora* sp. CNQ-490	AZUM00000000.1	GCF_000527075.1	Contig	4.94	71.1	25	25	4,518	613,253	3	Marine/Lake
*Saccharomonospora* sp. CUA-673	MKKE00000000.1	GCF_001942305.1	Contig	5.42	70.0	85	85	5,219	122,768	14	Marine/Lake

*^1^NCBI BioProject ID.*

### Phylogenetic and Phylogenomic Analyses

Phylogenetic trees based on the 16S rRNA gene sequences extracted from the annotated genomes or retrieved from GenBank/EMBL/DDBJ databases were constructed using the neighbor-joining (Saitou and Nei, 1987), maximum-parsimony (Fitch, 1971) and maximum-likelihood (Felsenstein, 1981) algorithms in MEGA X software (Kumar et al., 2018). The distance matrix was corrected using the Jukes-Cantor model of DNA evolution (Jukes and Cantor, 1969). Branch support was assessed by 1,000 bootstrap *pseudo*-replicates (Felsenstein, 1985). For phylogenomic tree construction only amino acidic sequences of single-copy core genes shared by all the analyzed genomes were used, as described elsewhere (de la Haba et al., 2019). Selection of those common orthologous genes (OGs) was done by finding the reciprocal best matches between pairs of genome sequences after an all-versus-all Blast search as implemented in the Enveomics collection toolbox (Rodriguez-R and Konstantinidis, 2016). Subsequently, selected OGs were aligned by using Muscle (Edgar, 2004), concatenated and employed for approximately maximum-likelihood phylogenomic tree reconstruction with FastTreeMP v.2.1.8 (Price et al., 2010) where branch confidence was evaluated using the Shimodaira-Hasegawa test (Shimodaira and Hasegawa, 1999).

### Comparative Genomic Analyses

Measures of similarity between genome sequences were achieved by using different algorithms to calculate Overall Genome Relatedness Indexes (OGRI) values, i.e., Orthologous Average Nucleotide Identity (OrthoANI), estimated using the OrthoANI-usearch Tool (Yoon et al., 2017), digital DNA-DNA hybridization (dDDH), determined using the Genome-to-Genome Distance Calculator (GGDC) (formula 2) (Meier-Kolthoff et al., 2013), and Average Amino-acid Identity (AAI), calculated using AAI calculator (Rodriguez-R and Konstantinidis, 2016).

Pan-genome analysis was carried out using the Enveomics tool (Rodriguez-R and Konstantinidis, 2016) to cluster CDSs in orthologous and singleton gene clusters, as indicated in section “Phylogenetic and Phylogenomic Analyses.” Pan-genome visualization was performed using Anvi’o (Eren et al., 2015). Flower plot displaying the core, accessory, and strain-specific genes was drawn in R using the ‘plotrix’ package. The characteristic curves showing the pan-genome and the core-genome evolution at the genus level were depicted using the Pan-Genome Profile Analyze Tool (PanGP) (Zhao et al., 2014) with distance guide (DG) sampling algorithm.

Synteny among the genomes of strains of the genus *Saccharomonospora* was studied after rearranging the draft genome scaffolds to a related reference genome using the Mauve Contig Mover functionality (Rissman et al., 2009). Pairwise comparison between ordered genomes was achieved by Blastn search (*e*-value ≤ 10−^3^) and synteny plots were displayed using Easyfig v.2.2.3 (Sullivan et al., 2011). Pan- and core-genome calculations were performed with the Enveomics package (Rodriguez-R and Konstantinidis, 2016).

### Biosynthetic Gene Cluster (BGC) Analysis

Location and identification of BGCs within genomes of *Saccharomonospora* was accomplished with the help of antiSMASH 5.0 web server (Blin et al., 2019), which uses Hidden Markov Models and rules based-detection to identify a broad array of BGCs, including those encoding PKS and NRPS. AntiSMASH detection strictness level was set to “relaxed.” In order to detect if the identified BGCs were enriched in the core- or accessory-genomes, we performed a nucleotide Blast search of the reverse translated protein pertaining to the core- and dispensable-genomes, respectively, against the BGCs from each of the analyzed genomes, and the corresponding Blast alignment length was used to calculate the fraction of BGCs spanned by core and accessory genes, respectively.

Since large BGCs (such as PKS, NRPS and hybrid clusters) may be split across several scaffolds/contigs when analyzing incomplete genomes leading to miscalculation of the predicted number of BGCs by antiSMASH, we used the NaPDoS (Natural Product Domain Seeker) tool (Ziemert et al., 2012) to connect PKS, NRPS or hybrid clusters divided into several scaffolds/contigs. BGC domains harbor the genetic signature of their historical relative and thus scaffolds/contigs containing pieces of one gene cluster are likely to phylogenetically clade together. Therefore, detection of PKS-derived ketosynthase domains (KS) and NRPS-derived condensation domains (C) with NaPDoS software, followed by alignment of the identified domain sequences with Muscle (Edgar, 2004) and phylogenetic reconstruction with Mega X software (Kumar et al., 2018) allow us to narrow down a more accurate number of PKS/NRPS/hybrid clusters present in fragmented next-generation sequencing assemblies. If a BGC was in the middle of a scaffold/contig (i.e., has sequence before and after the region antiSMASH identified), it is considered complete; otherwise it was marked as a partial cluster. If KS or C domains on different scaffolds/contigs were sister taxa in the domain-based trees (Supplementary Figure 1), the BGCs on the two or more scaffolds/contigs were considered part of one cluster and the corresponding scaffolds/contigs were joined together.

The total length of the prospective BGC was also taken into consideration to determine the most probably number of PKS and NRPS clusters. For each genome, the sum of the lengths of all clusters (partial and complete) within a category (PKS or NRPS) was divided by the average length of all the complete clusters into this category. The resulting measure is the expected number of PKS or NRPS clusters based on an average length specific to each genome (Supplementary Table 1). These estimates support the joining of clusters using NaPDoS and domain-based phylogenetic trees (Supplementary Figure 1).

Circular display of the corrected antiSMASH results was carried out using Circos Table Viewer v.0.63-9 visualization software (Krzywinski et al., 2009).

In order to predict the association of the detected BGCs with the production of known or novel bioactive compounds, the PKS, NRPS and hybrid clusters matches localized with antiSMASH were further screened with Blast search within the MIBiG database (Kautsar et al., 2020).

### Phenotypic Characterization

Comparative phenotypic features of strains *S. piscinae* KCTC 19743^T^ and *Saccharomonospora* sp. LRS4.154 were determined as described elsewhere (Tseng et al., 2018).

## Results

### *Saccharomonospora* Strain Grouping

A total of 19 genome sequences affiliated to members of the genus *Saccharomonospora* were retrieved from NCBI GenBank (18 genomes) and from JGI (1 genome) databases, 12 of which belong to type strains of species or subspecies within this genus, and the remaining seven were not affiliated to any particular species or subspecies (Table 1). The draft genome of *Saccharomonospora iraqiensis* subsp. *iraqiensis* IQ-H1^T^ was named as *Actinopolyspora iraqinesis* IQ-H1^T^ in GenBank database but it was also included in this study since both names are homotypic synonyms. Genome assembly varied between contig, scaffold, chromosome and complete genome level, with six out of the genomes assembled as a single scaffold or contig (Table 1). *Saccharomonospora* genomes have a size ranging from 3.69 Mb to 5.97 Mb, with a DNA G + C content of 67.3–71.3 mol% and contain between 3,656 and 5,664 protein-coding genes (Table 1).

The recovered genomes were grouped into four different sets according to their isolation source and to their NaCl requirements for growing (Table 2):

**TABLE 2:** Grouping of *Saccharomonospora* strains under study according to their isolation source and NaCl requirements for growing.

**Strain**	**Isolation source**	**NaCl range (optimum),% (w/v)**	**Group**
*Saccharomonospora amisosensis* DSM 45685^T^	Deep marine sediment in Black Sea (Turkey)	0–10	Marine/Lake
*Saccharomonospora marina* XMU15^T^	Ocean sediment of the East China Sea	0–5 (0–3)	
*Saccharomonospora piscinae* KCTC 19743^T^	Fishpond sediment in Taiwan	0–8 (5)	
*Saccharomonospora* sp. LRS4.154	Laguna del Rosario in Oaxaca (Mexico)	0–8 (5)	
*Saccharomonospora* sp. CNQ-490	Marine sediment in San Diego (United States)	∼2.5	
*Saccharomonospora* sp. CUA-673	Marine sponge in San Diego (United States)	∼2.5	
*Saccharomonospora halophila* 8^T^	Marsh soil in Kuwait	10–30 (10)	Moderately halophilic terrestrial
*Saccharomonospora iraqiensis* subsp. *iraqiensis* IQ-H1^T^	Extremely saline soil in Iraq	5–20 (10–15)	
*Saccharomonospora iraqiensis* subsp. *paurometabolica* YIM 90007^T^	Soil sample from the Xinjiang Province (China)	5–20 (10)	
*Saccharomonospora saliphila* YIM 90502^T^	Muddy soil Karnataka Province (India)	0–20 (10)	
*Saccharomonospora azurea* NA-128^T^	Soil sample from Sichuan (China)	≤ 7	Terrestrial
*Saccharomonospora azurea* SZMC 14600	Soil from China	≤ 7	
*Saccharomonospora cyanea* NA-134^T^	Soil samples from Sichuan (China)	≤ 10	
*Saccharomonospora glauca* K62^T^	Moldy hay, soil, compost, and manure from Germany	≤ 7	
*Saccharomonospora xinjiangensis* XJ-54^T^	Soil in Xinjiang (China)	0	
*Saccharomonospora* sp. 31sw	Soil from Iran	3	
*Saccharomonospora viridis* DSM 43017^T^	Manure, compost, overheated fodder, soil, lake sediments, peat	≤ 3	Clinical
*Saccharomonospora viridis* JCM 3315			
*Saccharomonospora viridis* ATCC 33517

• Marine/lake: strains isolated from marine environments or lakes and with optimal growth at or lower than 5% (w/v) NaCl.
• Moderately halophilic terrestrial: strains recovered from terrestrial hypersaline habitats and with optimal growth around 10% (w/v) NaCl.
• Terrestrial: strains from soil samples and manure growing between 0 and 10% (w/v) NaCl.
• Clinical: strains of the species *S. viridis*, the sole taxon in the genus associated to diseases.

### Taxophylogenomic Assessment

**FIGURE 1:**
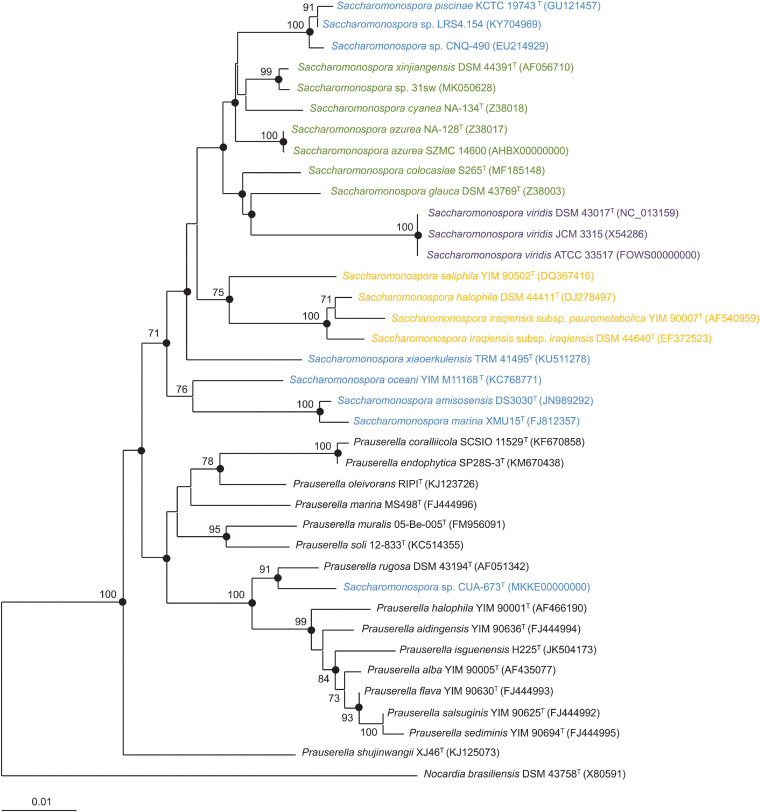
Neighbor-joining phylogenetic tree based on the 16S rRNA gene sequence comparison of members of the genus *Saccharomonospora* and the most closely related genus *Prauserella*. Bootstrap values ≥ 70% (based on 1,000 *pseudo*-replicates) are shown above the branches. Strains of *Saccharomonospora* were colored according to the group they belong to (blue for marine/lake, orange for moderately halophilic terrestrial, green for terrestrial and purple for clinical). Filled circles indicate clusters that were also recovered using maximum-parsimony and maximum-likelihood algorithms. *Nocardia brasiliensis* DSM 43758^T^ was used as an outgroup. Bar, 0.01 changes per nucleotide position.

The 16S rRNA gene sequence analysis was employed to infer the evolutionary relationships among the members of the currently described species of the genus *Saccharomonospora*, as well as other *Saccharomonospora* strains unassigned to any of the existing species (Figure 1). The closest related genus *Prauserella* was also included as a reference. The two genera were clearly separated according to this 16S rRNA gene-based phylogeny. All the unassigned *Saccharomonospora* strains were grouped into one of the described species of *Saccharamonospora*, with the exception of the strain *Saccharomonospora* sp. CUA-673, which clustered with the type strain of *Prauserella rugosa* and, therefore, seems to be misnamed. Sequence similarities for reference non-type or unnamed strains of *Saccharomonospora* with respect to the most closely related type strain were always above 99.5%, that is, beyond the threshold value for circumscribing prokaryotic species (98.65%) (Kim et al., 2014). Again, the only exception was *Saccharomonospora* sp. CUA-673, which shared less than 96.0% 16S rRNA gene sequence similarity with respect to the *Saccharomonospora* type strains but showed 98.7% sequence similarity to the type strain of *Prauserella rugosa*, indicating that it is closely related to that species. Therefore, no putative novel species of *Saccharomonospora* might be proposed after 16S rRNA gene sequence phylogenetic analysis. On the other hand, some type strains that clustered together based on 16S rRNA gene phylogeny showed a sequence similarity above the mentioned threshold value, particularly, *S. amisosensis* vs *S. marina* and the group constituted by *S. iraqiensis* subsp. *iraqiensis*, *S. iraqiensis* subsp. *paurometabolica* and *S. halophila*. So, according to the 16S rRNA gene sequence results, the species within each of those clusters might be merged into a single taxon, but a more confident evolutionary study is required to confirm the 16S rRNA gene-based hypothesis.

**FIGURE 2:**
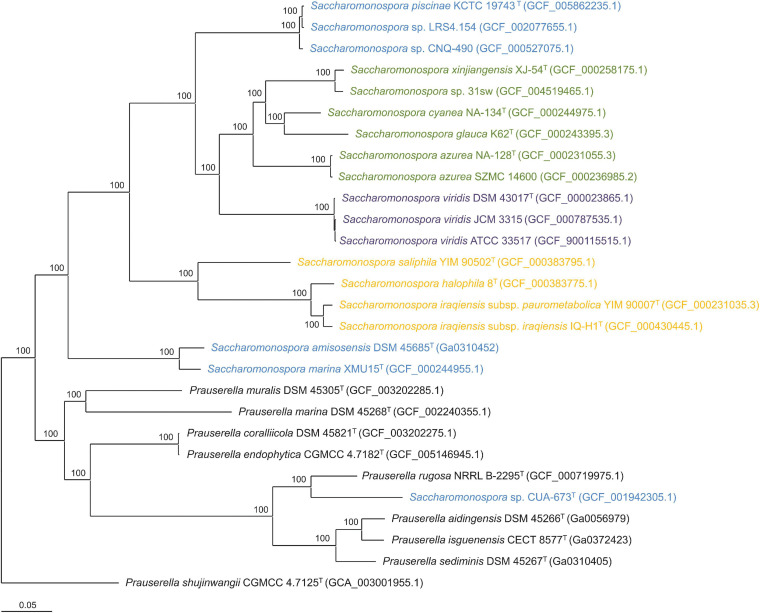
Approximately maximum-likelihood phylogenomic tree based on the concatenation of the translated sequence of the 876 single-copy genes shared by the members of the genera *Saccharomonospora* and *Prauserella* under study. Bootstrap values ≥ 70% (based on Shimodaira-Hasegawa-like local support) are shown above the branches. Strains of *Saccharomonospora* were colored according to the group they belong to (blue for marine/lake, orange for moderately halophilic terrestrial, green for terrestrial and purple for clinical). Bar, 0.05 changes per nucleotide position.

Phylogenomic trees that more reliably infer the evolutionary relationships among taxa can be obtained using the genome sequence information. In this particular case, we employed 876 concatenated amino acid sequences of the orthologous single-copy genes present in all the genomes under study (Figure 2). Although the clusters obtained in both trees were mostly in agreement, the phylogenomic tree showed a branch support of 100% in all bifurcations. This tree also points to a clear separation between *Saccharomonospora* and *Prauserella* genera, with probably no cryptic species of *Saccharomonospora* and with the need of transfer the strain *Saccharomonospora* sp. CUA-673 to the genus *Prauserella* (most likely as a new species given the long branch between this strain and the type strain of *Prauserella rugosa*). Similar to the 16S rRNA gene tree, the phylogenomic tree shows evidence for merging the species *S. amisosensis* – *S. marina*, on the one hand, and the species *S. iraqiensis* subsp. *iraqiensis* – *S. iraqiensis* subsp. *paurometabolica* – *S. halophila* on the other hand. Finally, according to the phylogenomic study, the species *Prauserella coralliicola* and *Prauserella endophytica* clustered very closely and most probably they constitute a single species, but this proposal is beyond the scope of the present manuscript.

We further analyzed the congruence between the clustering of *Saccharomonospora* strains in the phylogenetic/phylogenomic trees and the groups previously established based on the strain isolation source and NaCl requirements. Only clinical and moderately halophilic terrestrial isolates formed independent groups in the inferred phylogenies (Figures 1, 2). In the case of the clinical strains clustering was expected since the three clinical strains belonged to the same species and, in fact, two of them, namely *S. viridis* ATCC 33517 and *S. viridis* JCM 3315, are equivalent strains with different culture collection number. More interesting seems the grouping of moderately halophilic strains under the same clade, which suggests an environment-driven evolution of those strains to thrive in such extreme conditions. Although terrestrial strains were not clustered as a monophyletic branch according to the 16S rRNA gene phylogeny, they formed a monophyletic group in the phylogenomic tree (excepting the genome sequence of *S. colocasiae*), hinting at a common origin of those strains from soils, contaminants, and manure sources.

### Re-evaluation of the Species of *Saccharomonospora* Using Genome Sequence Distances

**FIGURE 3:**
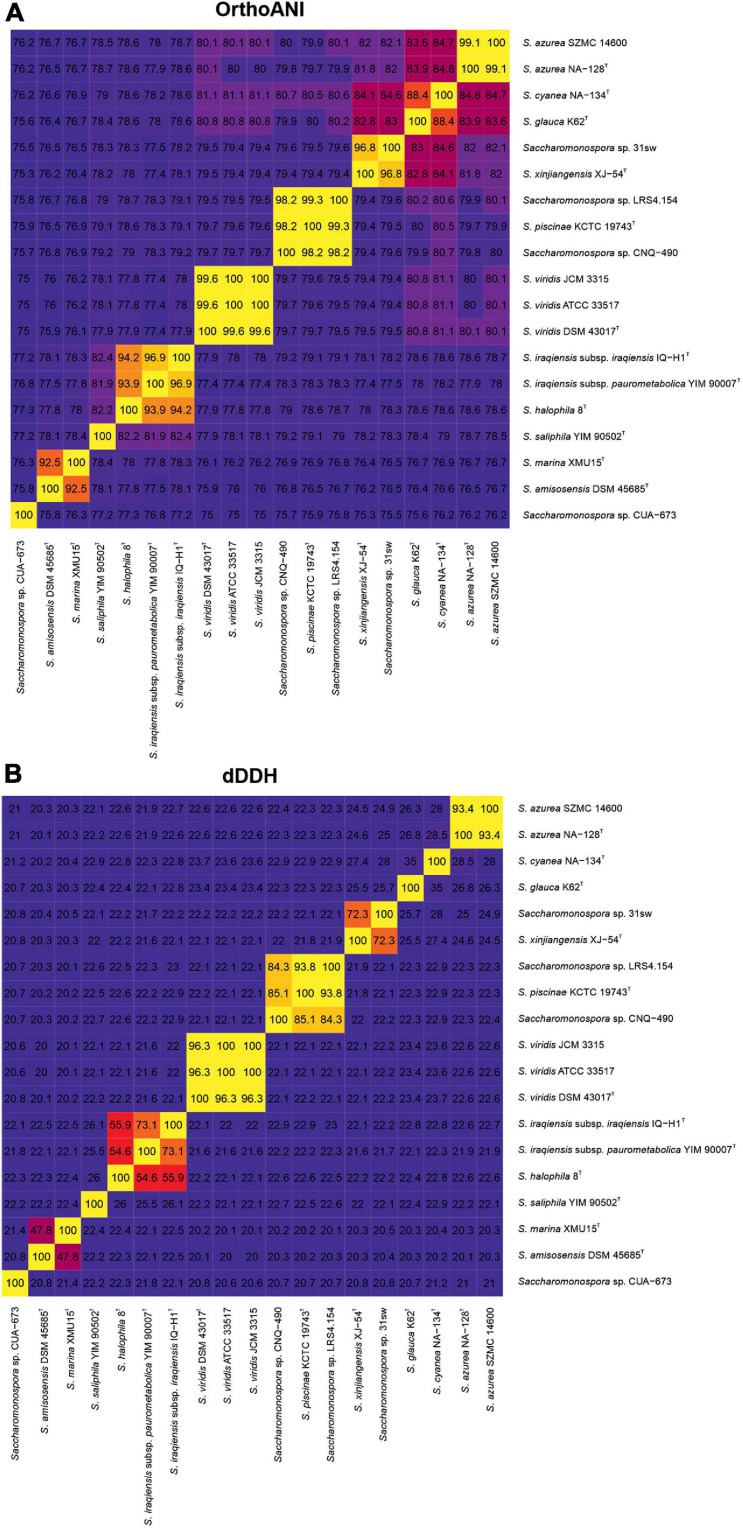
Heatmap of genome relatedness among members of the genus *Saccharomonospora* estimated by means of OrthoANI **(A)** and dDDH **(B)** values.

Similarity among genome sequences can be estimated by means of OGRI (Chun and Rainey, 2014). Although there are many algorithms to calculate OGRI values, the two most widely used for taxonomic purposes at species level are OrthoANI (Lee et al., 2016) and dDDH (Meier-Kolthoff et al., 2013). Gold standard for prokaryotic species delineation is still the wet-lab DNA-DNA hybridization (DDH), a widely considered tedious, laborious and error-prone method (Rosselló-Mora, 2006). A DDH cutoff value to unequivocally discriminate if two microbial genomes should be considered as the same or different species has been established at 70% (Wayne et al., 1987; Stackebrandt and Goebel, 1994). Nevertheless, OrthoANI and dDDH have been proposed as surrogates for DDH (Meier-Kolthoff et al., 2013; Lee et al., 2016), with well accepted threshold values for species delineation of approximately 95% (Konstantinidis and Tiedje, 2005a; Goris et al., 2007; Richter and Rossello-Mora, 2009; Chun and Rainey, 2014) and 70% (Auch et al., 2010), respectively. With regard to genus delineation, the most used OGRI value is AAI (Konstantinidis and Tiedje, 2005b), with a cutoff value about 65% (Konstantinidis et al., 2017). OrthoANI and dDDH values were calculated for all pairs of *Saccharomonospora* genomes (Figure 3) and for the pair *Saccharomonospora* sp. CUA-673 vs. *Prauserella rugosa* DSM 43194^T^ (OrthoANI 84.2%, dDDH 27.8%). Additionally, AAI values between members of *Saccharomonospora* and *Prauserella* were calculated.

As supported by phylogenetic/phylogenomic trees, AAI values indicated a good delineation of the genera *Saccharomonospora* and *Prauserella* with only a small overlap. AAI values within the *Saccharomonospora* genomes (excluding that of strain CUA-673) ranged from 71 to 100%, while values between the two genera varied from 67 to 76%. According to AAI values, strain CUA-673 was clearly a member of *Prauserella*, showing 66–82% AAI values with members of this genus in comparison to 65-66% AAI values with member of *Saccharomonospora*. On the other hand, OrthoANI and dDDH values demonstrated that no putative new species exist among the studied genomes of *Saccharomonospora* besides the already mentioned strain *Saccharomonospora* sp. CUA-673, which should be renamed as *Praseurella* sp. CUA-673 and constitute a novel species within this genus that may be proposed after a detailed taxonomic polyphasic approach (Tindall et al., 2010). The reference non-type strains *S. azurea* SZMC 14600 and *S. viridis* ATCC 33517 (= JCM 3315) are properly named, according to the OGRI values obtained with respect to the type strains of the corresponding species. Furthermore, the strains *Saccharomonospora* sp. LRS4.154 and *Saccharomonospora* sp. CNQ-490 are clearly additional strains of *S. piscinae* and should be renamed accordingly. Similarly, the strain *Saccharomonospora* sp. 31sw can be considered unequivocally a member of *S. xinjiangensis*, but the dDDH value slightly above the 70% cutoff between that strain (*Saccharomonospora* sp. 31sw) and the type strain of the species (*S. xinjiangensis* XJ-54^T^) and the 79–80% dDDH accepted value for subspecies boundaries (Meier-Kolthoff et al., 2014) suggest that strain *Saccharomonospora* sp. 31sw may constitute a novel separate subspecies within *S. xinjiangensis* taxon. Core-genome and 16S rRNA gene trees pointed to the need to merge the species *S. amisosensis* and *S. marina* into a single taxon, but OGRI analyses clearly rejected that idea. On the other hand, the taxonomic status of the group formed by *S. iraqiensis* subsp. *iraqiensis*, *S. iraqiensis* subsp. *paurometabolica* and *S. halophila* was not so clear since their OGRI results were close to the threshold values. *S. iraqiensis* subsp. *iraqiensis* and *S. iraqiensis* subsp. *paurometabolica* are doubtlessly different subspecies of the same species (supported by dDDH values of 73.1%, below the aforementioned subspecies cutoff). However, *S. halophila* fell within or close to the fuzzy zone [93–96% for OrthoANI (Rosselló-Móra and Amann, 2015), 60–70% for dDDH (Rosselló-Mora, 2006)] where the boundary of a species may not be clear. Therefore, *S. halophila* might be considered as a separate species or an additional novel subspecies of the species *S. iraqiensis*.

**FIGURE 4:**
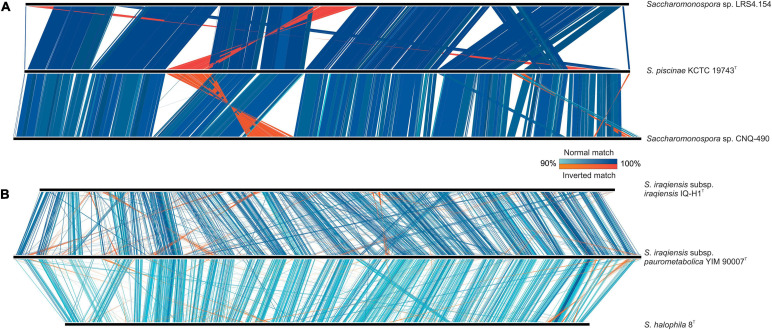
Synteny plot among highly conserved **(A)** and partially conserved **(B)** genomes of *Saccharomonospora*. Only matches with ≥ 500 bp alignment length and ≥ 90% identity are shown.

Spatial distribution of locally collinear blocks between two or more genomes (synteny) analysis can provide some clues about the evolutionary processes that lead to diversity, chromosomal dynamics, and rearrangement rates between species (Bhutkar et al., 2006). An approach to gain insight into the evolutionary distance between two species is to inspect the synteny of the genome sequences (Borriss et al., 2011). Here, we have applied the synteny study to elucidate suspicious clusters in the phylogenomic tree, particularly, the one formed by the two subspecies of *S. iraqiensis* and *S. halophila* 8^T^, using the cluster including *S. piscinae* KCTC 19743^T^, *Saccharomonospora* sp. LRS4.154 and *Saccharomonospora* sp. CNQ-490 as a reference for comparative purposes. The analysis revealed that the latter cluster possesses a very high level of synteny while the former was not so high (Figure 4). This might support the hypothesis that members of the second cluster are, indeed, members of the same species, while members of the first can be distinguished at species or subspecies level.

Going in depth in the *S. iraqiensis* subsp. *iraqiensis* – *S. iraqiensis* subsp. *paurometabolica* – *S. halophila* synteny analysis, although the genome coverage of locally collinear blocks is approximately the same between *S. iraqiensis* subsp. *iraqiensis* vs. *S. iraqiensis* subsp. *paurometabolica* and *S. iraqiensis* subsp. *iraqiensis* vs. *S. halophila*, the pairwise similarity between blocks is significantly higher for the pair *S. iraqiensis* subsp. *iraqiensis* – *S. iraqiensis* subsp. *paurometabolica* (Figure 4), endorsing the proposal to keep *S. halophila* as a different species while *S. iraqiensis* subsp. *iraqiensis* and *S. iraqiensis* subsp. *paurometabolica* would remain as subspecies of the same species.

### Almost Closed Pan- and Core-genomes of *Saccharomonospora*

**FIGURE 5:**
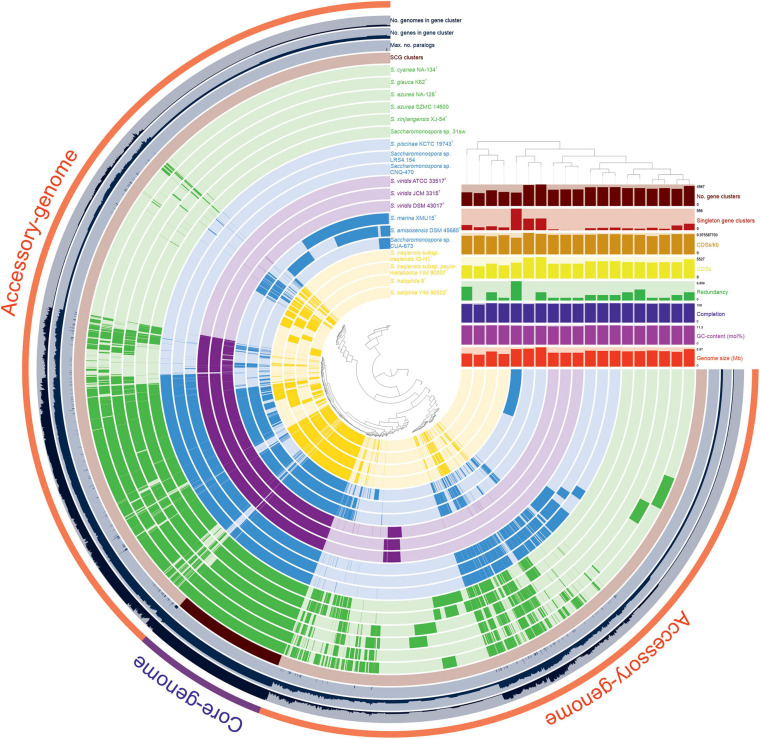
Comparative pangenome display of the 19 *Saccharomonospora* strains analyzed in this study. The inner layers represent individual genomes organized regarding their isolation source and their NaCl requirements for growing. In the layers, dark colors indicate the presence of a gene cluster and light color its absence. The core- (978 genes) and the accessory- (11,297 genes) genomes are indicated in purple and read, respectively, in the outmost layer.

The pan-genome defines the entire genomic repertoire of a given group of microorganisms and encodes for all possible lifestyles of its organisms, including the core genome, dispensable genome and strain-specific genes (Vernikos et al., 2015). The 79,484 protein CDSs detected in the 19 analyzed genomes of *Saccharomonospora* were grouped into 8,467 orthologous gene clusters (978 core genes and 7,489 dispensable genes) and 3,808 singletons (strain-specific) gene clusters, with a pan-genome constituted of 12,275 gene clusters (Figure 5, Supplementary Figure 2). This wide range difference between the core- and pan-genomes might be due to the large genome size of actinobacteria and the existence of a large number of accessory genes in the *Saccharomonospora* genomes, some of them related to BGCs. In fact, BGCs were enriched in the accessory-genome in comparison to the core-genome, with an average of 71.1% of BGC length spanned by accessory genes versus an average of 6.5% spanned by core genes.

**FIGURE 6:**
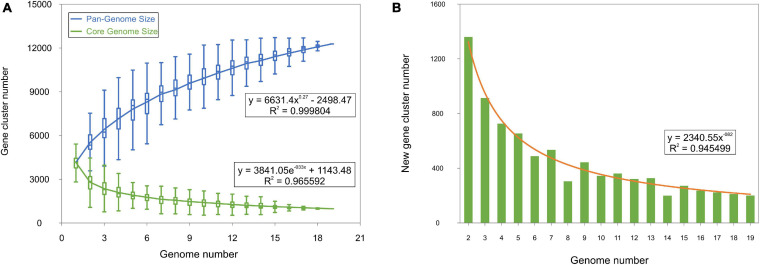
Pan- and core-genome size evolution within the genus *Saccharomonospora*. **(A)** Gene accumulation curves of the pan-genome (blue) and the core-genome (green). The curve is the least squares fit of the power law for the average values. **(B)** Number of new genes and fit curve (orange) with an increase in the number of *Saccharomonospora* genomes.

Compared to other actinobacteria, studies focused on the genus *Streptomyces* identified 15,404 (Wu et al., 2017), 23,672 (Tidjani et al., 2019), and 34,592 (Kim et al., 2015) gene clusters conforming the pan-genome of 9, 11, and 17 members, respectively, in this genus, while the core-genome decreased from 5,047 (when considering 9 streptomycetes) to 2,018 (for 17 streptomycetes) gene clusters, suggesting an open pan- and core-genomes. Nevertheless, the evolution of the pan- and core-genomes of the 19 *Saccharomonospora* genomes rapidly reaches a plateau (Figure 6), indicating almost closed pan- and core-genomes, which means that most of the natural variation has been captured.

### Secondary Metabolite Profiling

**FIGURE 7:**
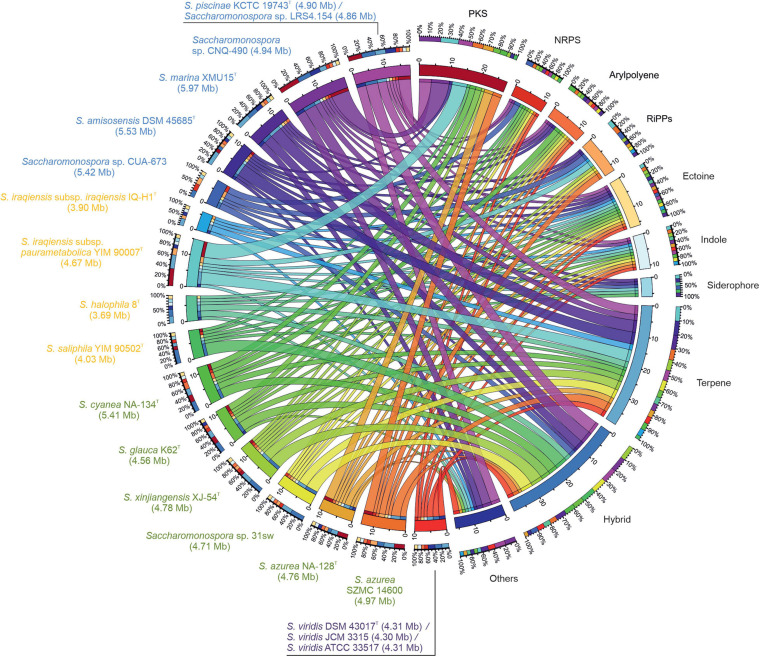
Circular diagram of *Saccharomonospora* BGCs diversity. Genomes of strains with identical BGCs pattern are collapsed in a single band. Each genome is represented by a different colored band (left half of the circle) that can be traced from the organism to the types of BGCs found in that genome (right half of the circle). The width of these bands indicates the number of BGCs of that type. The cluster types are also assigned colors that make up the outer ring next to each genome to easily see what portion of each genome is assigned to a specific BGC. Conversely, the outer ring next to the BGC categories show the proportion of that BGC attributed to each genome represented by the genome color. The RiPP category includes clusters identified as bacteriocin, lanthipeptide or lassopeptide. The Hybrid category includes gene clusters constituted by two or more BGC types. The “Others” category includes uncommon BGCs found in less than five genomes, i.e., betalactone, homoserine lactone, ladderane, linaridin and oligosaccharide. Strains of *Saccharomonospora* were colored according to the group they belong to (blue for marine/lake, orange for moderately halophilic terrestrial, green for terrestrial and purple for clinical) and the genome size is indicated in parentheses.

The genomes of members of the genus *Saccharomonospora* were analyzed for the presence of a broad array of secondary metabolites BGCs, including those encoding PKS, NRPS, terpenes, ectoine, indole, arylpolyene, siderophore and ribosomally synthesized and post-translationally modified peptides (RiPPs). The BGCs identified were further curated to determine which PKS, NRPS and hybrid partial clusters most likely belonged to the same gene cluster. In total numbers, the most abundant non-hybrid BGCs predicted in the studied actinobacterial genomes were terpene, followed by PKS and ectoine, although the hybrid BGCs were even more plentiful (Figure 7, Supplementary Tables 2, 3). Marine/lake and terrestrial strains possess on average the higher amount of putative BGCs (14.5 and 12.2, respectively), while clinic and moderately halophilic terrestrial ones contain slightly less (10 and 9.5, respectively). *Saccharomonospora* sp. CNQ-490 followed by *S. piscinae* KCTC 19743^T^ and *Saccharomonospora* sp. LRS4.154 (all three belonging to the same species as aforementioned) were, theoretically, the most prolific secondary metabolites producers with the higher number of identified BGCs (Figure 7, Supplementary Tables 2, 3). There were some types of secondary metabolites able to be produced only by specific strains/species, i.e., homoserine lactone (in the closely related strains *S. piscinae* KCTC 19743^T^, *Saccharomonospora* sp. LRS4.154 and *Saccharomonospora* sp. CNQ-490), lassopeptide (in the probably misnamed strain *Saccharomonospora* sp. CUA-673) and oligosaccharide (in the moderately halophilic strain *S. iraqiensis* subsp. *paurometabolica* YIM 90007^T^). It is also remarkable that no PKS or NRPS clusters were predicted for *S. marina* XMU15^T^, *S. iraqiensis* subsp. *iraqiensis* IQ-H1^T^ and *S. halophila* 8^T^ (Figure 7, Supplementary Tables 1–3).

Polyketide synthase, NRPS and hybrid PKS-NRPS clusters found by antiSMASH (Blin et al., 2019) were further searched using Blast tool against MIBiG database (Kautsar et al., 2020) in order to predict known and novel biomolecules that might putatively be synthesized by members of *Saccharomonospora*. In theory, BGCs for production of alkil-O-dihydrogeranyl-methoxyhydroquinones, mirubactin, sporolide, curamycin and taromycin are the most widely spread among the members of this genus, being present in four to seven genomes with at least 50% of genes showing similarity to the corresponding MIBiG cluster (Table 3). On the contrary, BGCs for the synthesis of JBIR-100, oxazolepoxidomycin A and saprolmycin could only be detected in one genome with more than 50% of genes showing similarity to a known MIBiG cluster. Other BGCs related to the production of acarviostatin I03/acarviostatin II03/acarviostatin III03/acarviostatin IV03, anthracimycin, apoptolidin, arsenopolyketides, desosamine, ebelactone, ECO-02301, enduracidin, friulimicin, indanomycin, kedarcidin, octacosamicin, PM100117/PM100118, rabelomycin, ralsolamycin, retimycin, sanglifehrin A, selvamicin, skyllamycin and tiancimycin were also present only in one genome, but sharing less than 50% of the genes with the registered MIBiG cluster (Table 3), which means that might be involved in the synthesis of a novel compound. Noticeably, for a specific type 1 PKS cluster identified in *Saccharomonospora* sp. CNQ-490, two NRPS and hybrid PKS-NRPS clusters in *Saccharomonospora* sp. CUA-673, and a type 3 PKS cluster in *S. iraqiensis* subsp. *paurometabolica* YIM 90007^T^ no matches to any predicted known product were found by cluster Blast search and, therefore, a more detailed study of these clusters as potential producers of new biomolecules might be interesting.

**TABLE 3:** Putative synthesis of known bioactive compounds by PKS, NRPS or hybrid BGCs of strains of *Saccharomonospora* based on Blast search against MIBiG database.

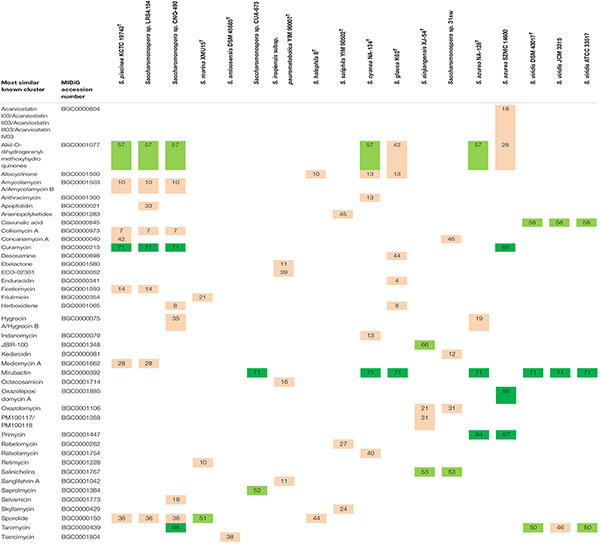

*Cell values indicate the percentage of genes within the MIBiG cluster showing similarity to the query sequence (color code: light orange, < 50% genes; green, 50–69% genes; dark green, 70–100% genes).*

## Discussion

Comparative 16S rRNA gene sequence analysis has been widely used to infer relationships within the genus *Saccharomonospora* (Kim, 2015), but despite its success in providing the phylogenetic backbone for the classification of *Actinobacteria* in the past, this gene possesses a low evolutionary rate, which means only a limited number of differential characters and, thus, may yield trees with many statistically unsupported branches (Klenk and Göker, 2010; Breider et al., 2014). Furthermore, this gene might be present in multiple divergent copies within a single genome and might have suffered horizontal gene transfer yielding to mistaken phylogenies (Větrovský and Baldrian, 2013; Papke et al., 2015). For those reasons, the use of the concatenated sequence of several single-copy housekeeping genes (an approach called Multilocus Sequence Analysis, MLSA) with a higher resolution was proposed to overcome these limitations (Gevers et al., 2005), but the produced phylogenies were limited to a certain number of genes. With the decreasing cost of genome sequencing and the ever-increasing prokaryotic genomes in public databases, phylogenetic trees may be substituted by phylogenomic trees inferred based on a large number of core single-copy protein-coding genes (Eisen and Fraser, 2003). In this study we have compared the 16S rRNA gene-based phylogeny of the genus *Saccharomonospora* with the evolutionary relationships obtained by using a phylogenomic approach. A previous study inferring trees from genome-scale data within the phylum *Actinobacteria* has been carried out (Nouioui et al., 2018) but, on the one hand, it included only 11 genomes of *Saccharomonospora* and, secondly, the phylogenomic tree was constructed using whole proteomes (instead of translated single-copy core genes) and distance-based clustering methods, which are not as robust as approximately maximum-likelihood-based methods (Price et al., 2010). In any case, the topology of the phylogenomic tree regarding the genus *Saccharomonospora* were consistent between the study of Nouioui et al. (2018) and the present research (Figure 2).

Here, we have studied in more detail all the 19 available genomes of *Saccharomonospora* using an approximately maximum-likelihood-based approach. Our results showed that 16S rRNA gene-based phylogenies reliably reflect the relationships among members of *Saccharomonospora*, with almost identical topologies between the phylogenetic and the phylogenomic trees. Therefore, despite all the drawbacks that have been described for 16S rRNA single gene trees, this approach is still quite accurate for the actinobacterial genus *Saccharomonospora* besides the modest tree branch support. The use of genomic data allowed us to unequivocally assign the strains *Saccharomonospora* sp. LRS4.154 and *Saccharomonospora* sp. CNQ-490 to the species *S. piscinae* (which makes it necessary to emend the description of the species as indicated below), the strain *Saccharomonospora* sp. 31sw to the species *S. xinjiangensis* and the strain *Saccharomonospora* sp. CUA-673 to the closest genus *Prauserella* as a putative new species which might be formally proposed after a detailed polyphasic study (that could not be conducted here since the strain CUA-673 is not publicly available in any culture collection). We have also confirmed that the strains *S. azurea* SZMC 14600 and *S. viridis* ATCC 33517 (= JCM 3315) are properly named. The taxonomic status of *S. iraqiensis* subsp. *iraqiensis*, *S. iraqiensis* subsp. *paurometabolica* and *S. halophila* remains unclear after several studies. Tang et al. proposed the transference of *S. iraqiensis* subsp. *iraqiensis* IQ-H1^T^ (at that time *Actinopolyspora iraqiensis* IQ-H1^T^) as a heterotypic synonym of *S. halophila* 8^T^ based on wet-lab DDH data (Tang et al., 2011). Later, using dDDH values and distance-based phylogenomic inference it was found that the strain IQ-H1^T^ was more closely related to *S. paurometabolica* YIM 90007^T^ and it was proposed the merger of both taxa as a single species, but separated as two different subspecies (Nouioui et al., 2018). Authors of this study considered that the name *Actinopolyspora iraqiensis* had priority over *S. paurometabolica*, which is true taking into account that the former name was validly published before the latter, and therefore they proposed the name *S. iraqiensis* to accommodate the two subspecies. Beyond nomenclature issues, the relationships among the three above mentioned taxa needed revision. In this study, we have hypothesized that *S. halophila* might constitute another subspecies of *S. iraqiensis* because of OrthoANI and dDDH values close to the transition zone of the species boundary. Nevertheless, synteny plots among those taxa demonstrated that locally collinear blocks possess higher similarity between *S. iraqiensis* subsp. *iraqiensis* and *S. iraqiensis* subsp. *paurometabolica* compared to those shared by *S. iraqiensis* subsp. *iraqiensis* and *S. halophila*. Therefore, our study supports a recent speciation event occurring in *S. halophila* or *S. iraqiensis* which may explain that the obtained OGRI values between both taxa fall close to the species threshold value. Finally, our analysis endorses the current status of the remaining species of *Saccharomonospora* as independent taxa.

In this study we also focused on the identification of biosynthetic gene clusters (BGCs) in the genome of the analyzed strains of *Saccharomonospora*. Only a few previous reports mining BGCs in this genus have been published so far (Yamanaka et al., 2014; Schorn et al., 2016; Reynolds et al., 2018; Kovács et al., 2020), which investigated the genomes of *Saccharomonospora* sp. CNQ-490, *Saccharomonospora* sp. CUA-673 (hereby putatively relocated to the genus *Prauserella*), and two strains of *S. azurea* in search of their secondary metabolic capacity. Compared to other genera of *Actinobacteria*, the genus *Saccharomonospora* showed a high degree of novelty and diversity of BGCs (Schorn et al., 2016). Our study, considering all the available *Saccharomonospora* genomes, supports the previous findings, with a wide BGC abundance in each genome ranging from 6 to 19 identified clusters corresponding to 18 different categories. Although it has been stated that the number and variety of BGC pathways generally increases as the size of the genome increases (Schorn et al., 2016), we have observed that this pattern does not properly fit to the genus *Saccharomonospora*, where the Pearson’s correlation value between number of BGCs and genome size is rather weak (0.38). Moreover, strains belonging to the same species can contain large differences, such as *S. iraqiensis* subsp. *paurometabolica* YIM 90007^T^ which almost triples *S. iraqiensis* subsp. *iraqiensis* IQ-H1^T^ in amount of BGCs. The three strains pertaining to the species *S. piscinae* showed the highest abundance and variety of BGCs, with a total of 18 to 19 clusters corresponding to 11 different categories. However, attention must be paid not only to the abundance and diversity, but also to particular BGCs contained only in some strains. That is the case of the homoserine lactone cluster, occurring only in *S. piscinae* strains, or lassopeptide and oligosaccharide clusters, found exclusively in *Saccharomonospora* (*Prauserella*) sp. CUA-673 and *S. iraqiensis* subsp. *paurometabolica*, respectively.

Polyketide synthase (PKS) and non-ribosomal peptide synthetase (NRPS) are multi-domain megasynthases involved in the biosynthesis of a remarkable amount of biological active compounds clinically valuable as anti-microbial, anti-fungal, anti-parasitic, anti-tumor and immunosuppressive agents (Cane and Walsh, 1999) and, therefore, require special attention. Detailed analysis of PKS, NRPS and hybrid clusters, and their comparison to available databases may provide clues to predict the product that a microorganism can synthetize. BGC mining conducted in this research demonstrated that a total of 40 different putative bioactive products might be produced by PKS, NRPS and hybrid clusters of *Saccharomonospora* (Table 3). Some compounds can theoretically be made by several strains of this genus, while others are specific to a particular strain. Most important, several BGCs showed no match to any already discovered compound or had a low similarity to known molecules, in particular clusters related to the synthesis of acarviostatin I03/acarviostatin II03/acarviostatin III03/acarviostatin IV03, allocyclinone, amycolamycin A/amycolamycin B, anthracimycin, apoptolidin, arsenopolyketides, collismycin A, concanamycin A, desosamine, ebelactone, ECO-02301, enduracidin, ficellomycin, friulimicin, herboxidiene, hygrocin A/hygrocin B, indanomycin, kedarcidin, mediomycin A, octacosamicin, oxazolomycin, PM100117/PM100118, rabelomycin, ralsolamycin, retimycin, sanglifehrin A, selvamicin, skyllamycin and tiancimycin, which shared less than 50% of the genes with the BGCs in the MIBiG database (Table 3), and they are probably involved in the synthesis of new bioactive compounds with promising clinical and biotechnological applications. In spite of the large genomic potential of *Saccharomonospora* BGCs to synthetize bioactive molecules, a literature search shows that only a few bioactive compounds (primycin, taromycin, and saccharomonopyrones A-C) have been isolated so far from members of the genus *Saccharomonospora* (Yamanaka et al., 2014; Yim et al., 2017; Reynolds et al., 2018; Kovács et al., 2020) and, therefore, a more comprehensive screening to isolate more biomolecules that can be synthetized by members of this genus should be carried out. Primycin is known to be produced only by strains of the species *S. azurea* (Kovács et al., 2020), which coincides with our genomic data (Table 3). Taromycin is reported to be isolated from the marine *Saccharomonospora* sp. CNQ-490 (Yamanaka et al., 2014; Reynolds et al., 2018), although our genomic analysis indicated that members of the species *S. viridis* might also produce taromycin-like compounds (Table 3). Besides, the strain *Saccharomonospora* sp. CNQ-490 can yield three α-pyrones (namely saccharomonopyrones A-C) (Yim et al., 2017), but those biomolecules were not detected by the *in silico* analysis because they are not included into the MIBiG database.

## Conclusion

Genome-scale data of *Saccharomonospora* strains can be used to elucidate evolutionary relationships among microorganisms of this genus and to unequivocally assign strains into species. Based on the results of this manuscript we, hereby, propose the emended description of the species *S. piscinae* as indicated below. This genus has been revealed as an important source of a wide variety of biosynthetic gene clusters, which may be responsible for the production of novel non-yet-discovered bioactive molecules with potential application to biotechnology and biomedicine.

### Emended Description of *Saccharomonospora piscinae* Tseng et al. (2018)

The description is as given before (Tseng et al., 2018), with the following amendment. The aerial mycelium is pale green to greenish gray or black. Oval spores have smooth or rugose surface. Olive green soluble pigments may be produced. Halotolerant or moderate halophile. Growth occurs between 0 to 20% (w/v) NaCl and 20 to 55°C. Gelatin may be liquefied. Menaquinone MK-8(H_2_) may be present as minor component. DNA G + C content is 70.6–71.0 mol% (genome).

Strains LRS4.154 (= CECT 9353 = DSM 105201) and CNQ-490, isolated from saline soil of Laguna El Rosario, Oaxaca, Mexico, and from a deep-sea sediment sample 2 km west of the Scripps pier, La Jolla, CA, United States, respectively, are additional reference strains of this species.

## Data Availability Statement

The genome sequences analyzed in this study can be found in the GenBank and Joint Genome Institute databases under the accession numbers shown in Table 1.

## Author Contributions

NR-D, RH, BV-G, CS-P, HS-T, and AV did the conceptualization. NR-D and RH did the methodology, formal analysis, data curation, writing-original draft preparation, and visualization. BV-G, CS-P, and SA-C did the validation. NR-D, RH, and CS-P did the investigation. NR-D, RH, and AV did the resources. NR-D, RH, BV-G, CS-P, SA-C, HS-T, and AV did the writing – review and editing. HS-T and AV did the supervision. CS-P and AV did the project administration. NR-D, CS-P, and AV did the funding acquisition. All authors have read and agreed to the published version of the manuscript.

## Conflict of Interest

The authors declare that the research was conducted in the absence of any commercial or financial relationships that could be construed as a potential conflict of interest.
