# Supplementary material for: Taxogenomic and Comparative Genomic Analysis of the Genus Saccharomonospora Focused on the Identification of Biosynthetic Clusters PKS and NRPS
Source: Front Microbiol. 2021 Mar 11;12:603791. doi: 10.3389/fmicb.2021.603791 (PMC7990883; doi:10.3389/fmicb.2021.603791)
Supplement: Supplementary file 1 [file Data_Sheet_1.pdf]

Supplementary Material

Taxogenomic and Comparative Genomic Analysis of the Genus *Saccharomonospora* Focused on the Identification of Biosynthetic Clusters PKS and NRPS

Ninfa Ramírez-Durán[1,2,†], Rafael R. de la Haba[2,†], Blanca Vera-Gargallo[2], Cristina Sánchez-Porro[2], Scarlett Alonso-Carmona[1], Horacio Sandoval-Trujillo[3], Antonio Ventosa[2,*]

[1]Faculty of Medicine, Autonomous University of the State of Mexico, 50180 Toluca, Mexico
[2]Department of Microbiology and Parasitology, Faculty of Pharmacy, University of Sevilla, 41012 Sevilla, Spain
[3]Department of Biological Systems, Metropolitan Autonomous University-Xochimilco, 04960 Mexico City, Mexico

† These authors contributed equally to this work

*** Correspondence:**
Antonio Ventosa
ventosa@us.es

1 Supplementary Figures and Tables

1.1 Supplementary Figures

A

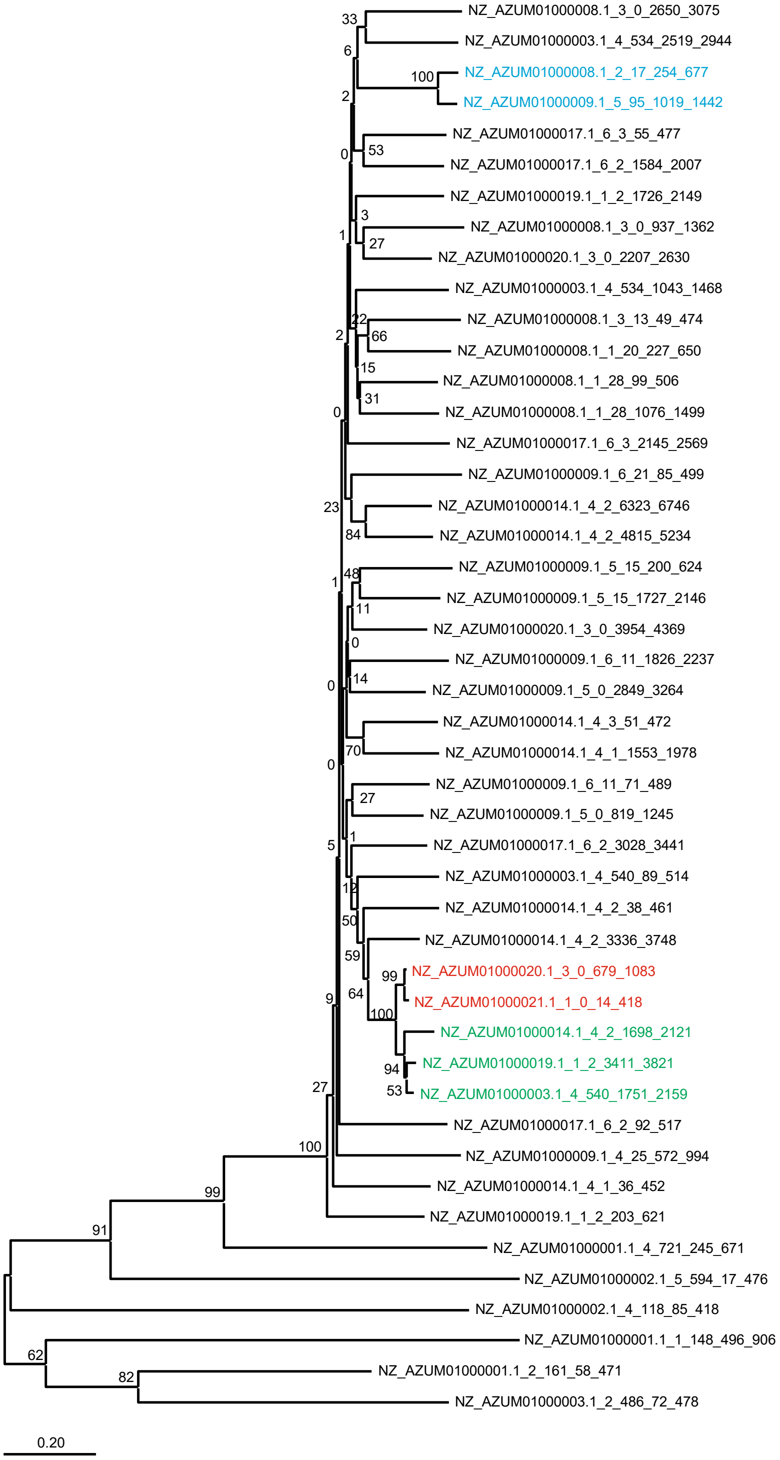

B

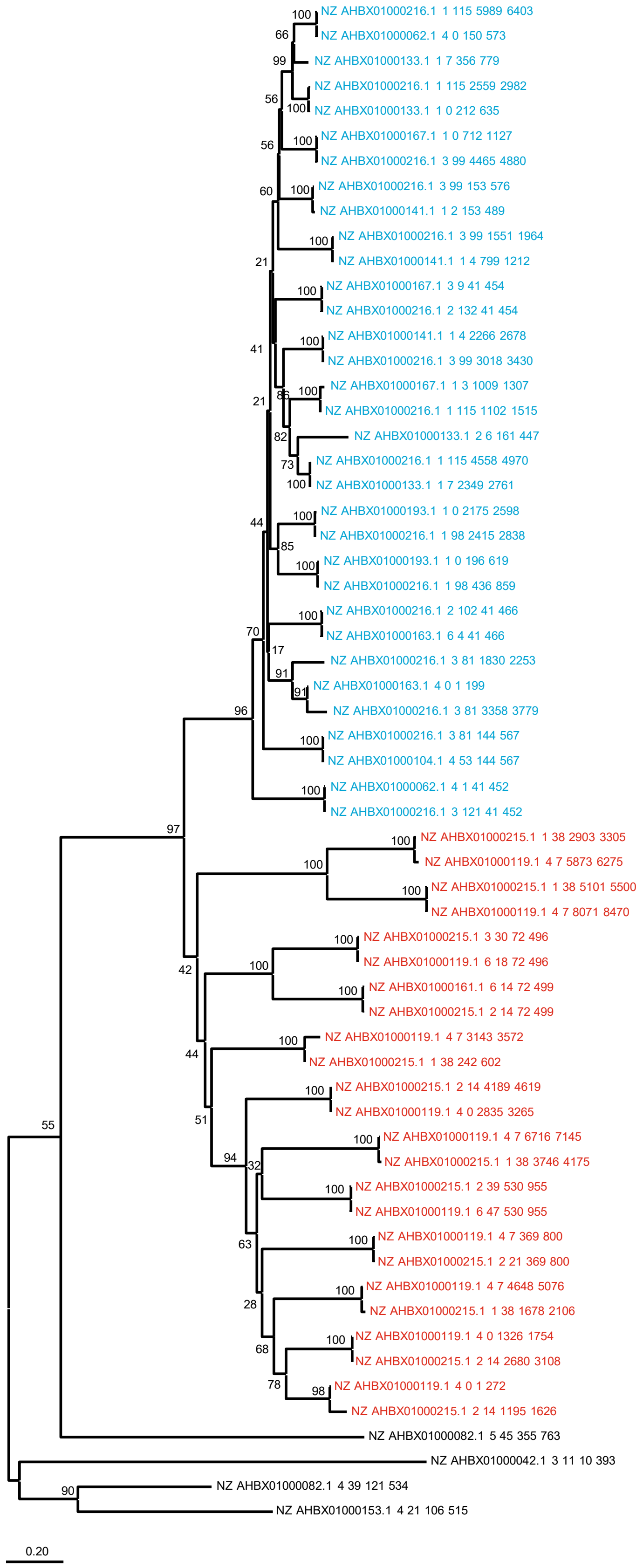
NZ AHBX01000216.1 1 115 5989 6403
NZ AHBX01000062.1 4 0 150 573
NZ AHBX01000133.1 1 7 356 779
NZ AHBX01000216.1 1 115 2559 2982
NZ AHBX01000133.1 1 0 212 635
NZ AHBX01000167.1 1 0 712 1127
NZ AHBX01000216.1 3 99 4465 4880
NZ AHBX01000216.1 3 99 153 576
NZ AHBX01000141.1 1 2 153 489
NZ AHBX01000216.1 3 99 1551 1964
NZ AHBX01000141.1 1 4 799 1212
NZ AHBX01000167.1 3 9 41 454
NZ AHBX01000216.1 2 132 41 454
NZ AHBX01000141.1 1 4 2266 2678
NZ AHBX01000216.1 3 99 3018 3430
NZ AHBX01000167.1 1 3 1009 1307
NZ AHBX01000216.1 1 115 1102 1515
NZ AHBX01000133.1 2 6 161 447
NZ AHBX01000216.1 1 115 4558 4970
NZ AHBX01000133.1 1 7 2349 2761
NZ AHBX01000193.1 1 0 2175 2598
NZ AHBX01000216.1 1 98 2415 2838
NZ AHBX01000193.1 1 0 196 619
NZ AHBX01000216.1 1 98 436 859
NZ AHBX01000216.1 2 102 41 466
NZ AHBX01000163.1 6 4 41 466
NZ AHBX01000216.1 3 81 1830 2253
NZ AHBX01000163.1 4 0 1 199
NZ AHBX01000216.1 3 81 3358 3779
NZ AHBX01000216.1 3 81 144 567
NZ AHBX01000104.1 4 53 144 567
NZ AHBX01000062.1 4 1 41 452
NZ AHBX01000216.1 3 121 41 452
NZ AHBX01000215.1 1 38 2903 3305
NZ AHBX01000119.1 4 7 5873 6275
NZ AHBX01000215.1 1 38 5101 5500
NZ AHBX01000119.1 4 7 8071 8470
NZ AHBX01000215.1 3 30 72 496
NZ AHBX01000119.1 6 18 72 496
NZ AHBX01000161.1 6 14 72 499
NZ AHBX01000215.1 2 14 72 499
NZ AHBX01000119.1 4 7 3143 3572
NZ AHBX01000215.1 1 38 242 602
NZ AHBX01000215.1 2 14 4189 4619
NZ AHBX01000119.1 4 0 2835 3265
NZ AHBX01000119.1 4 7 6716 7145
NZ AHBX01000215.1 1 38 3746 4175
NZ AHBX01000215.1 2 39 530 955
NZ AHBX01000119.1 6 47 530 955
NZ AHBX01000119.1 4 7 369 800
NZ AHBX01000215.1 2 21 369 800
NZ AHBX01000119.1 4 7 4648 5076
NZ AHBX01000215.1 1 38 1678 2106
NZ AHBX01000119.1 4 0 1326 1754
NZ AHBX01000215.1 2 14 2680 3108
NZ AHBX01000119.1 4 0 1 272
NZ AHBX01000215.1 2 14 1195 1626
NZ AHBX01000082.1 5 45 355 763
NZ AHBX01000042.1 3 11 10 393
NZ AHBX01000082.1 4 39 121 534
NZ AHBX01000153.1 4 21 106 515
0.20

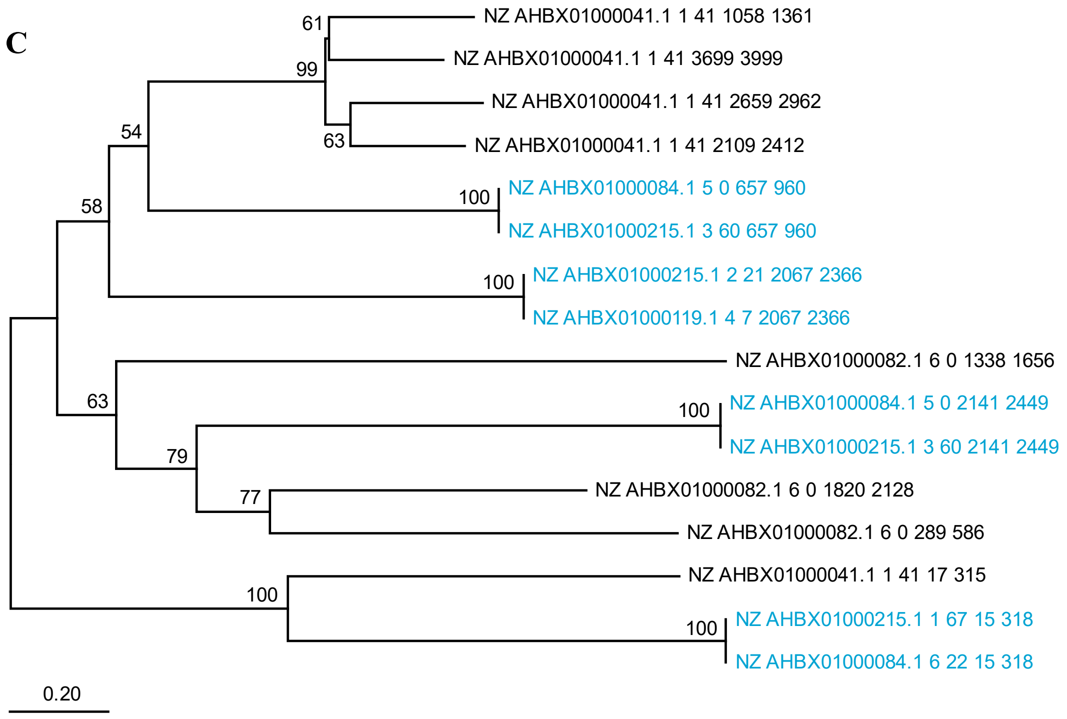

Supplementary Figure S1. Phylogenetic trees based on KS or C domains detected by NaPDoS software used to connect PKS, NRPS and hybrid clusters split onto two or more scaffolds/contigs. (A) *Saccharomonospora* sp. CNQ-490 KS domain-based tree. (B) *S. azurea* SZMC 14600 KS domain-based tree. (C) *S. azurea* SZMC 14600 C domain-based tree. Scaffolds/contigs marked in the same color were joined together.

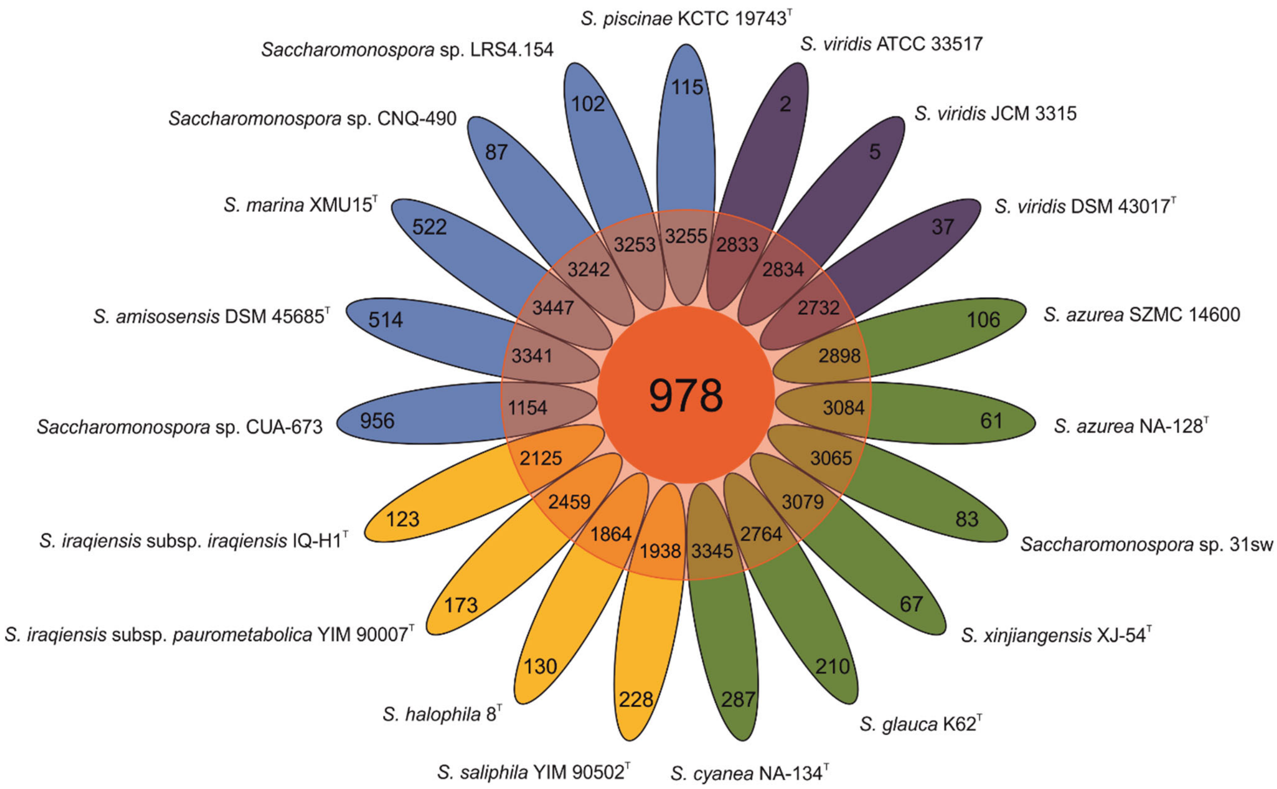

Supplementary Figure S2. Flower plot showing the core (in the center), dispensable (in the annulus), and strain-specific (in the petals) genes of the 19 *Saccharomonospora* strains. Different colors indicate different *Saccharomonospora* groups according to their isolation source and their NaCl requirements for growing

1.2 Supplementary Tables

Supplementary Table S1. Expected number of PKS and NRPS clusters specific to each genome based on an average length. For each genome, the sum of the lengths of all PKS or NRPS clusters was divided by the average length of all the complete PKS or NRPS clusters.

Strain	PKS					NRPS				
	# complete PKS	length complete PKS	PKS average length	Total PKS length	Estimated PKS number (rounded up)	# complete NRPS	length complete NRPS	NRPS average length	Total NRPS length	Estimated NRPS number (rounded up)
***S. piscinae* KCTC 19743^T**	3	159,431	53,144	172,993	4	1	43,071	43,071	43,071	1
***Saccharomonospora* sp. LRS4.154**	3	148,145	49,382	161,723	4	1	43,071	43,071	43,071	1
***Saccharomonospora* sp. CNQ-490**	3	159,422	53,141	311,072	6	1	43,071	43,071	43,071	1
***S. marina* XMU15^T**	0	0	0	0	0	0	0	0	0	0
***S. amisosensis* DSM 45685^T**	1	45,499	45,499	45,499	1	0	0	0	0	0
***Saccharomonospora* sp. CUA-673**	0	0	0	0	0	2	103,485	51,743	103,485	2
***S. iraqiensis* subsp. *iraqiensis* IQ-H1^T**	0	0	0	0	0	0	0	0	0	0
***S. iraqiensis* subsp. *paurometabolica* YIM 90007^T**	3	117,397	39,132	188,203	5	0	0	0	0	0
***S. halophila* 8^T**	0	0	0	0	0	0	0	0	0	0
***S. saliphila* YIM 90502^T**	1	46,537	46,537	46,537	1	1	38,827	38,827	38,827	1
***S. cyanea* NA-134^T**	2	113,560	56,780	113,560	2	1	68,169	68,169	68,169	1

***S. glauca* K62[T]**	2	113,566	56,783	113,566	2	1	48,760	48,760	48,760	1
***S. xinjiangensis* XJ-54[T]**	1	113,596	113,596	113,596	1	1	52,738	52,738	52,738	1
***Saccharomonospora* sp. 31sw**	0	0	0	0	0	1	53,120	53,120	53,120	1
***S. azurea* NA-128[T]**	3	211,528	70,509	211,528	3	0	0	0	0	0
***S. azurea* SZMC 14600**	2	179,101	89,551	255,595	3	1	61,714	61,714	61,714	1
***S. viridis* DSM 43017[T]**	0	0	0	0	0	1	91,321	91,321	91,321	1
***S. viridis* JCM 3315**	0	0	0	0	0	1	91,321	91,321	91,321	1
***S. viridis* ATCC 33517**	0	0	0	0	0	1	91.855	91,855	91,855	1

Supplementary Table S2. Number of pathways for each category as output by antiSMASH 5.0 web server. Strains are colored by total pathway number, with blue = low, green = medium, red = high amount of clusters. Each hybrid cluster (marked in fold face) is detailed in the last column. For PKS, NRPS and hybrid clusters joined by NaPDoS approach, the final predicted number of putative clusters is given in parentheses.

**Strain**	**Total BGCs**	**Type 1 PKS**	**Type 2 PKS**	**Type 3 PKS**	**NRPS**	**Arylpolyene**	**Bacteriocin (RiPP)**	**Lanthipeptide (RiPP)**	**Lassopeptide (RiPP)**	**Ectoine**	**Indole**	**Siderophore**	**Terpene**	**Hybrid**	**Betalactone (Others)**	**Homoserine_lactone (Others)**	**Ladderane (Others)**	**Linaridin (Others)**	**Oligosaccharide (Others)**	**Hybrid types**
S. piscinae **KCTC 19743^{T}**	18	2	1	1	1	1	0	0	0	1	1	0	3	**4**	0	1	0	2	0	NRPS+T1PKS, T1PKS+T3PKS, betalactone+NRPS, siderophore+T1PKS
Saccharomonospora **sp. LRS4.154**	18	2	1	1	1	1	0	0	0	1	1	0	3	**4**	0	1	0	2	0	NRPS+T1PKS, siderophore+T1PKS, betalactone+NRPS, T1PKS+T3PKS
Saccharomonospora **sp. CNQ-490**	23 (19)	7 (4)	1	1	1	1	0	0	0	1	1	0	2	**5 (4)**	0	1	0	2	0	NRPS+T1PKS, LAP+Bacteriocin, NRPS+betalactone, T3PKS+NRPS+T1PKS+siderophore
S. marina **XMU15^{T}**	13	0	0	0	0	1	0	1	0	1	0	1	4	**3**	1	0	1	0	0	NRPS+bacteriocin+betalactone, lanthipeptide+NRPS+T1PKS, T1PKS+NRPS
S. amisosensis **DSM 45685^{T}**	11	1	0	0	0	1	0	1	0	1	0	1	4	**1**	1	0	0	0	0	betalactone+hglE-KS
Saccharomonospora **sp. CUA-673**	8	0	0	0	2	0	0	1	1	1	0	1	0	**2**	0	0	0	0	0	T2PKS+oligosaccharide, NRPS+T1PKS
S. iraqiensis **subsp.** ***iraqiensis*** **IQ-H1^{T}**	6	0	0	0	0	0	0	1	0	1	1	0	2	**0**	1	0	0	0	0	
S. iraqiensis **subsp.** ***paurometabolica*** **YIM 90007^{T}**	15	4	0	1	0	0	1	1	0	1	1	1	4	**0**	0	0	0	0	1	

S. halophila **8^{T}**	8	0	0	0	0	0	0	0	0	1	1	1	2	**2**	0	0	0	1	0	T1PKS+NRPS, T3PKS+T2PKS+NRPS
S. saliphila **YIM 90502^{T}**	9	1	0	0	1	0	0	0	0	1	0	1	1	**4**	0	0	0	0	0	ladderane+arylpolyene, terpene+siderophore, NRPS+T3PKS+T2PKS, other+T3PKS+T2PKS
S. cyanea **NA-134^{T}**	12	0	1	1	1	2	0	0	0	1	1	0	2	**3**	0	0	0	0	0	NRPS+T1PKS, NRPS+arylpolyene, NRPS+transAT-PKS
S. glauca **K62^{T}**	11	0	1	1	1	1	0	0	0	1	0	0	2	**3**	0	0	0	1	0	other+NRPS+oligosaccharide, NRPS+T1PKS, betalactone+NRPS
S. xinjiangensis **XJ-54^{T}**	13	1	0	0	1	1	1	0	0	1	1	0	3	**4**	0	0	0	0	0	NRPS+T1PKS, T1PKS+T3PKS, ladderane+arylpolyene, T2PKS+transAT-PKS+NRPS
Saccharomonospora **sp. 31sw**	12	0	0	0	1	1	1	0	0	1	1	0	3	**4**	0	0	0	0	0	T3PKS+T1PKS, T1PKS+transAT-PKS+NRPS+T2PKS, NRPS+arylpolyene, ladderane+arylpolyene
S. azurea **NA-128^{T}**	11	2	0	1	0	1	1	0	0	1	1	0	2	**1**	1	0	0	0	0	T2PKS+NRPS+transAT-PKS+T1PKS
S. azurea **SZMC 14600**	22 (14)	8 (2)	0	1	2 (1)	1	2	0	0	1	1	0	3	**3 (2)**	0	0	0	0	0	transAT-PKS+NRPS+T1PKS, T2PKS+NRPS
S. viridis **DSM 43017^{T}**	10	0	0	0	1	1	0	0	0	1	1	0	2	**2**	0	0	2	0	0	blactam+T2PKS, T3PKS+NRPS
S. viridis **JCM 3315**	10	0	0	0	1	1	0	0	0	1	1	0	2	**2**	0	0	2	0	0	blactam+T2PKS, T3PKS+NRPS
S. viridis **ATCC 33517**	10	0	0	0	1	1	0	0	0	1	1	0	2	**2**	0	0	2	0	0	NRPS+T3PKS, blactam+T2PKS
**TOTAL**	240 (232)	28 (22)	5	8	15 (14)	15	6	5	1	19	14	6	46	**49 (48)**	4	3	7	8	1	

Supplementary Table S3. Input table used to create the circular diagram (Figure 7). Genomes containing identical BGCs pattern were collapsed. PKS, NRPS and hybrid clusters that could be connected by NaPDoS approach are included in this table. All types of PKS clusters were also collapsed into one category. Bacteriocin, lanthipeptide and lassopeptide are collapsed into the category "RiPPs" (Ribosomally synthesized and Post-translationally modified Peptides). All minor categories present in less than five genomes (i.e. betalactone, homoserine lactone, ladderane, linaridin and oligosaccharide) were collapsed into the "Others" category. Strains of *Saccharomonospora* were colored according to the group they belong to (blue for marine/lake, orange for halophilic terrestrial, green for terrestrial and purple for clinical).

**Strain**	**Estimated genome size (Mb)**	**Total PKS**	**NRPS**	**Arylpolyene**	**RiPP**	**Ectoine**	**Indole**	**Siderophore**	**Terpene**	**Hybrid**	**Others**
S. piscinae **KCTC 19743^T/ *Saccharomonospora* sp. LRS4.154**	4.90/4.86	4	1	1	0	1	1	0	3	4	3
Saccharomonospora **sp. CNQ-490**	4.94	6	1	1	0	1	1	0	2	4	3
S. marina **XMU15^T**	5.97	0	0	1	1	1	0	1	4	3	2
S. amisosensis **DSM 45685^T**	5.53	1	0	1	1	1	0	1	4	1	1
Saccharomonospora **sp. CUA-673**	5.42	0	2	0	2	1	0	1	0	2	0
S. iraqiensis **subsp.** ***iraqiensis*** **IQ-H1^T**	3.90	0	0	0	1	1	1	0	2	0	1
S. iraqiensis **subsp.** ***paurometabolica*** **YIM 90007^T**	4.67	5	0	0	2	1	1	1	4	0	1
S. halophila **8^T**	3.69	0	0	0	0	1	1	1	2	2	1
S. saliphila **YIM 90502^T**	4.03	1	1	0	0	1	0	1	1	4	0
S. cyanea **NA-134^T**	5.41	2	1	2	0	1	1	0	2	3	0
S. glauca **K62^T**	4.56	2	1	1	0	1	0	0	2	3	1
S. xinjiangensis **XJ-54^T**	4.78	1	1	1	1	1	1	0	3	4	0
Saccharomonospora **sp. 31sw**	4.71	0	1	1	1	1	1	0	3	4	0

***S. azurea* NA-128[T]**	4.76	3	0	1	1	1	1	0	2	1	1
***S. azurea* SZMC 14600**	4.97	3	1	1	2	1	1	0	3	2	0
***S. viridis* DSM 43017[T]/*S. viridis* JCM 3315/ *S. viridis* ATCC 33517**	4.31/4.30/4.31	0	1	1	0	1	1	0	2	2	2
